# Nature images are more visually engaging than urban images: evidence from neural oscillations in the brain

**DOI:** 10.3389/fnhum.2025.1575102

**Published:** 2025-06-17

**Authors:** Amy S. McDonnell, Sara B. LoTemplio, Emily E. Scott, David L. Strayer

**Affiliations:** ^1^Department of Psychology, University of Utah, Salt Lake City, UT, United States; ^2^Department of Human Dimensions of Natural Resources, Colorado State University, Fort Collins, CO, United States; ^3^Department of Psychological Sciences, Vermont State University, Burlington, VT, United States

**Keywords:** attention restoration theory, nature, EEG, frontal theta, parietal alpha, visual engagement, cognitive demand

## Abstract

**Introduction:**

Attention Restoration Theory posits that urban environments place high demand on our attentional systems, which can fatigue over time and lead to impairments in performance. On the contrary, natural environments are proposed to visually engage our attention but in a less demanding way, allowing for the recuperation of attentional resources and subsequent improvements in attentional performance. However, the neural mechanisms underlying these varying attentional demands remain poorly understood. The current study utilized electroencephalography (EEG) to explore attention-related brain activity when individuals view images of nature and urban environments.

**Methods:**

In a between-subjects design, 58 participants viewed 10-min of either nature or urban images while brain activity was recorded. Frequency-domain measures of parietal alpha and frontal theta were extracted from the raw EEG data to quantify visual engagement and cognitive demand, respectively.

**Results:**

Participants that viewed nature images displayed significantly lower parietal alpha power than participants that viewed urban images, suggesting nature scenes are more visually engaging than urban scenes. Participants that viewed nature images also displayed trends toward lower frontal theta power than participants that viewed urban images, suggesting that nature scenes are less cognitively demanding to process, though this effect was not statistically significant. Lastly, nature images were self-reported to be more restorative than urban images.

**Discussion:**

Taken together, these results suggest that natural scenes are visually engaging, but not in a cognitively demanding fashion. This aligns with Attention Restoration Theory and prior literature suggesting that nature scenes engage effortless, involuntary attention while allowing effortful attention to rest and recover.

## Introduction

The rapidly growing field of environmental neuroscience explores the influence of various physical environments on human cognition and brain functioning (Berman and Bratman, 2024). There is particular interest in the distinction between natural and urban environments and their influence on human cognition, with mounting evidence suggesting that immersion in nature differentially influences brain activity related to stress (Sudimac et al., 2022; Sudimac and Kühn, 2022), mood (Bratman et al., 2015; Norwood et al., 2019), and attention (LoTemplio et al., 2020; McDonnell and Strayer, 2024a, 2024b; McDonnell et al., 2025). Understanding the neurological mechanisms that underlie nature’s benefits is essential for developing evidence-based health interventions, designing environments that promote well-being, and addressing public health challenges in an increasingly urbanized world.

Various neuroimaging methods hold promise for studying the influence of natural and urban environments on brain functioning both in laboratory settings and in real-world environments. Researchers have leveraged functional magnetic resonance imaging (fMRI) and functional near-infrared spectroscopy (fNIRS) to observe the brain’s response to natural and urban stimuli. Both fMRI and fNIRS measure brain activity by detecting underlying changes in blood flow. This body of work has documented promising changes in the hemodynamic response associated with walking in nature (Bratman et al., 2015; Sudimac et al., 2022), viewing images of nature (Kühn et al., 2021; Tang et al., 2017; Yamashita et al., 2021), watching videos of nature (Yu et al., 2017), and listening to nature soundscapes (Gould van Praag et al., 2017; Stobbe et al., 2024). However, both fMRI and fNIRS measure neural activity *indirectly* through hemodynamic responses, which are inherently slower than *direct* measures of neuronal activity. These delayed hemodynamic responses introduce a methodological limitation when studying how rapid processes—such as fluctuations in attention—unfold in real time.

### Electroencephalography

Electroencephalography (EEG) provides a direct measure of the electrical activity of the brain by detecting postsynaptic voltage fluctuations generated by neuronal activity (Cohen, 2017; Jackson and Bolger, 2014). Specifically, EEG records the synchronous activity of large groups of neurons through electrodes placed on the scalp. EEG is a particularly promising tool for the field of environmental neuroscience for several reasons (Grassini, 2024). First, it can capture the rapid dynamics of neuronal activity with a temporal resolution on the order of milliseconds. This high precision allows researchers to track changes in brain activity in response to environmental stimuli in near real time. Furthermore, EEG is portable and relatively cost-effective. This portability enables the study of brain activity in ecologically-valid, outdoor environments rather than confining experiments to inside the laboratory (e.g., Hopman et al., 2020; LoTemplio et al., 2020; McDonnell et al., 2025).

Event-related potential (ERP) components and resting-state oscillatory activity are two distinct aspects of brain activity captured in EEG data that have been quantified in the environmental neuroscience literature. ERP components are momentary changes in brain activity in response to specific events in the environment (Luck and Kappenman, 2013). They are considered phase-locked, meaning they consistently appear at the same time relative to a stimulus. Resting oscillatory activity, on the other hand, represents the brain’s ongoing, spontaneous rhythms when *not* actively engaged in a task (Cohen, 2014). Thus, these oscillations are not time-locked to any external event but rather represent the brain’s intrinsic activity. Oscillatory activity is measured in frequency bands [e.g., theta (4–8 Hz), alpha (8–12 Hz), beta (12–30 Hz)] that are linked to brain states like relaxation, engagement, or arousal. Both ERP components and resting oscillatory activity provide valuable insights into brain function but serve different purposes in research and clinical applications.

Numerous studies in the environmental neuroscience literature have demonstrated the strengths of using EEG (both ERP components and resting oscillatory activity) to understand the influence of exposure to nature on brain activity. Researchers have indexed changes in task-related ERP components associated with either immersion in nature (LoTemplio et al., 2020; McDonnell et al., 2025; McDonnell and Strayer, 2024a) or the viewing of nature images (Collins et al., 2025; Grassini et al., 2019; Mahamane et al., 2020; McDonnell et al., 2025). While these studies have yielded crucial insights into real-time changes in cognitive processes while participants are performing cognitive tasks, they are unable to provide insights into fluctuations in cognition that occur *outside* the parameters of a task. Measures of oscillatory activity, which are the focus of the current study, overcome this limitation by allowing researchers to record brain activity in the absence of a task, providing greater insight into the brain’s spontaneous response to different environments.

### Attention restoration theory

This study, like much of the prior EEG work in the environmental neuroscience literature, is grounded in Attention Restoration Theory (ART; Kaplan, 1995), a theoretical framework that proposes one potential mechanism by which nature positively influences human cognition. ART posits that natural environments are restorative because they alleviate the attentional demands that are placed on us in our modern, urban environments. Specifically, urban environments are thought to place high demand on our attentional systems, forcing us to sort through many incoming stimuli at once, selecting relevant information while ignoring distracting information (Kaplan, 1995). These constant mental gymnastics can place strain on our limited attentional resources and cause mental fatigue over time (Boksem et al., 2005), leading to detriments in self-regulation and task performance (Kaplan and Berman, 2010).

On the other hand, ART proposes that natural environments are less demanding to process (Kaplan, 1995). In nature, the mind is often not required to concentrate as intensely or perform cognitively demanding tasks. Instead, nature consists of ‘soft’ stimuli—like the sound of birds chirping, the rustling of leaves, or the flow of water. These ‘softer’ stimuli in nature are thought to facilitate a process of restoration by engaging involuntary attention—attention that is not effortful but is rather effortlessly engaged (James, 1891). This, ART proposes, allows the brain to recover from the strain caused by prolonged periods of focusing on tasks that require high mental effort, thereby improving cognitive performance, mood, and overall well-being (Kaplan, 1995).

Importantly, ART proposes that for natural environments to be restorative, four key qualifications must be met. First, the environment must be *compatible* with the individual, meaning that it aligns with the individual’s needs, preferences, and goals. For example, someone seeking relaxation may benefit from a tranquil garden or a quiet forest walk, while someone interested in physical activity might find restoration in a more dynamic setting like a bike path in a park. Second, *being away* is another essential element of ART, referring to the idea that for an environment to be truly restorative, individuals need to feel mentally removed or disconnected from the stressors and demands of their everyday lives. This allows people to break free from the mental strain that comes with work, obligations, and daily responsibilities. Being away does not always necessarily mean physically escaping to a remote location. It can also mean a mental shift—moving from a state of focused, goal-directed attention to one of relaxed, open awareness. Third, the environment must have *extent*. This refers to the idea that a restorative environment must feel expansive and immersive, offering enough richness to engage the mind without overwhelming it. The environment should have sufficient depth and variety, such as diverse landscapes or features, to encourage exploration in many directions. The fourth qualification of *‘soft fascination’* is characterized by effortless engagement with stimuli that gently hold attention, such as the patterns on leaves, the sound of a flowing stream, or the slow movement of clouds. Unlike the intense focus required for tasks in daily life, these types of stimuli draw our attention without depleting it.

### Parietal alpha power

This fourth qualification of ‘*soft fascination*’ lends itself to the cognitive process of visual engagement, such that natural environments are thought to contain visually engaging yet non-demanding stimuli. Alpha oscillations (8–12 Hz) distributed over parietal and occipital regions of the brain (i.e., visual cortices) can be quantified as a neural measure of visual engagement (Clayton et al., 2018; Peylo et al., 2021; Rana and Vaina, 2014; Romei et al., 2008) and thus can be used to assess how visually engaged an individual is with their environment. Specifically, parietal alpha power is *inversely* related to visual engagement such that in eyes-open conditions when the visual system is actively processing the surrounding visual field, parietal alpha power is typically low (Foxe and Snyder, 2011). Conversely, when the eyes are closed and there is no visual input, parietal alpha power is high (Goldman et al., 2002). This pattern supports the interpretation of alpha as a visual inhibitory mechanism, wherein increased alpha activity reflects the suppression of visual cortex excitability (Jensen and Mazaheri, 2010; Romei et al., 2008). Alpha activity is proposed to arise from a thalamic-cortical loop, with higher parietal alpha power linked to the inhibition of visual awareness (Foxe and Snyder, 2011). In contrast, reductions in parietal alpha power occur when visual attention is directed toward visually engaging stimuli in the environment (Kirschfeld, 2005).

While numerous studies in the environmental neuroscience literature have quantified the influence of exposure to natural stimuli on alpha oscillations over various scalp locations (e.g., Chen et al., 2020; Grassini et al., 2022; Rhee et al., 2023), Hopman et al. (2020) specifically quantified *parietal* alpha power—or alpha power over visual cortices—as a neural signature of visual engagement. This work measured changes in parietal alpha power associated with immersion in nature compared to control conditions in an urban environment, revealing a significant decrease in parietal alpha power during immersion in real nature as participants sat on a riverbank and viewed the nature scene in front of them. Given parietal alpha oscillations are inversely related to visual engagement, this suggests that the nature scene was more visually engaging than the control, laboratory scenes, perhaps aligning with the idea of ‘*soft fascination*’. The current study expands upon this work by exploring whether viewing *images* of nature has this same effect on parietal alpha power as viewing real nature scenes.

### Frontal theta power

The other key aspect of ART is that nature scenes are visually engaging *without being cognitively demanding*. It is thought that this property of nature allows for the rest and recovery of neural circuits related to cognitive demand that are often over-engaged in urban environments.

Theta oscillations (4–8 Hz) distributed over frontal regions of the brain are a reliable measure of cognitive demand (Chikhi et al., 2022). Source localization studies show that frontal theta oscillations reflect dorsal anterior cingulate cortex activity (dACC; Gevins et al., 1997), a subcortical region in the brain that is highly implicated in effortful attention (Shenhav et al., 2016; Silton et al., 2010; Weissman et al., 2005). The dACC, in connection with the prefrontal cortex, is thought to manage incoming stimuli and allocate attention depending on task goals, particularly in scenarios requiring focus amidst competing stimuli or distractions (Shenhav et al., 2016). Frontal theta oscillations show a strong positive correlation with cognitive demand, such that frontal theta power increases with greater task difficulty and higher working memory demands (Gevins et al., 1997; Jensen and Tesche, 2002). In large, frontal theta oscillations reflect the dynamic interaction between the dACC and related brain networks during attention allocation processes, offering a window into the neural mechanisms that support effortful attention. Importantly, studies of resting oscillatory activity have shown that higher frontal theta power at rest is related to declines in performance on cognitive tasks, likely due to increased cognitive load at rest (Tan et al., 2024). Thus, ART might predict that nature lowers frontal theta power at rest, thereby reducing demands on attention which translate into more efficient use of attentional resources for demanding tasks later.

Few studies in the environmental neuroscience literature have quantified changes in frontal theta power associated with exposure to nature. McDonnell and Strayer (2024b) quantified resting frontal theta power in 92 participants before and after they were randomized to walk for 40 min in either nature or an urban environment. They found that frontal theta power was significantly greater after the urban walk compared to the nature walk, suggesting that the urban environment placed higher demand on attentional systems (indicated by greater frontal theta power measured immediately after the urban walk) whereas the nature walk did not place as much demand on attention. However, this study relied on pre- and post-walk recordings of frontal theta power, making it difficult to conclude how frontal theta power (and the underlying neural networks associated with cognitive demand) is modulated *during* immersion in these environments. The current study fills this gap by recording frontal theta oscillations during the actual viewing of nature versus urban images rather than relying on a pre-post intervention design.

### Current study

Grounded in ART, the current study explores neural oscillations related to visual engagement (i.e., parietal alpha power) and cognitive demand (i.e., frontal theta power) during the viewing of nature and urban images. There is a pre-existing and rather expansive literature that explores whether nature images can restore attention, with “restoration” often conceptualized as improved performance on computer-based behavioral tasks that engage attention (e.g., Berman et al., 2008; Collins et al., 2025; McDonnell et al., 2025). Therefore, rather than directly testing whether nature images improve subsequent task performance, this study aims to understand the neural processes engaged during the actual viewing of the nature images, under the premise that these neural mechanisms may underlie and facilitate subsequent attention restoration.

We are not the first to quantify neural oscillations during the viewing of nature versus urban images. However, previous research often adopts an exploratory approach by examining multiple oscillatory patterns across multiple regions of the scalp (e.g., Grassini et al., 2019). While this method provides a broad and comprehensive perspective, it can make the findings challenging to interpret within established theoretical frameworks like ART. Instead, we adopt an *a priori*, hypothesis-driven approach, focusing specifically on parietal alpha power (as a signature of visual engagement) and frontal theta power (as a signature of cognitive demand) in an effort to isolate specific neural mechanisms most relevant to the theory. This approach is grounded in the principles of ART, with the goal of uncovering the neurobiological mechanisms that underpin ART’s proposition that natural stimuli engage attention in a way that is visually engaging but not cognitively demanding.

In the current study, participants were randomized to view 10 min of either nature or urban images while the EEG signal was continuously recorded. Power in the parietal alpha (8–12 Hz) and frontal theta (4–8 Hz) frequency bands were extracted from the EEG signal as neural signatures of visual engagement and cognitive demand, respectively. At the end of testing, participants completed the Perceived Restorativeness Scale as a self-report index of how restorative they considered the image set they viewed to be. In alignment with prior literature, we hypothesized that:

*H*_1_: The nature group would display lower parietal alpha power than the urban group, suggesting nature images are more visually engaging than urban images.

*H*_2_: The nature group would display lower frontal theta power than the urban group, suggesting nature images are less cognitively demanding to process compared to urban images.

*H*_3_: The nature group would score higher on the Perceived Restorativeness Scale than the urban group, suggesting nature images are subjectively more restorative than urban images.

## Methods and materials

This research complied with the APA Code of Ethics and was approved by the University of Utah Institutional Review Board (IRB_ 00129483). Informed consent was obtained from each participant and the reported methods were performed in accordance with relevant guidelines and regulations of this institution.

### Participants

Participants (*N* = 61; 74% female, 26% male, 0% non-binary) between the ages of 18 and 44 (*M* = 24.69, *SD* = 5.48) were recruited through the University of Utah Department of Psychology SONA Participant Pool and via flyers in the greater Salt Lake City community. 78% of participants identified as White/Non-Hispanic, 15% identified as Asian, 3% identified as White/Asian, 2% identified as Hispanic/Latino, and 2% identified as Black/African American. Community participants were compensated $20 for their time and SONA Participant Pool participants were granted two research credits applicable to their general psychology course. An *a priori* power analysis using G*Power indicated that a total sample size of 52 participants (26 per condition) would be needed to detect a medium between-groups effect (Cohen’s *d* = 0.40) with 80% statistical power (Cohen, 1988). We collected data from 61 participants to account for anticipated data loss, ensuring that even after data loss we would remain above the target sample size of 52 participants.

### Procedure

Upon arrival to the lab, participants completed the IRB-approved consent process. The research team then set up the EEG electrodes on the participants’ scalp. Once the EEG electrodes were properly secured to the scalp and signal quality was deemed acceptable, participants were randomized to passively view either 10-min of nature images or 10-min of urban images on a computer screen while EEG data were continuously recorded. Following image viewing, participants completed three, computer-based cognitive tasks whose results are reported elsewhere (Collins et al., 2025; McDonnell et al., 2025). Participants then completed the Perceived Restorativeness Scale via paper and pencil in which they reported the extent to which they considered the image set they viewed (either nature or urban) to be restorative. The procedure took 2 h to complete, at which point the EEG electrodes were removed and the participant was either paid or granted research credit for their involvement.

### Stimulus presentation

**Figure 1:**
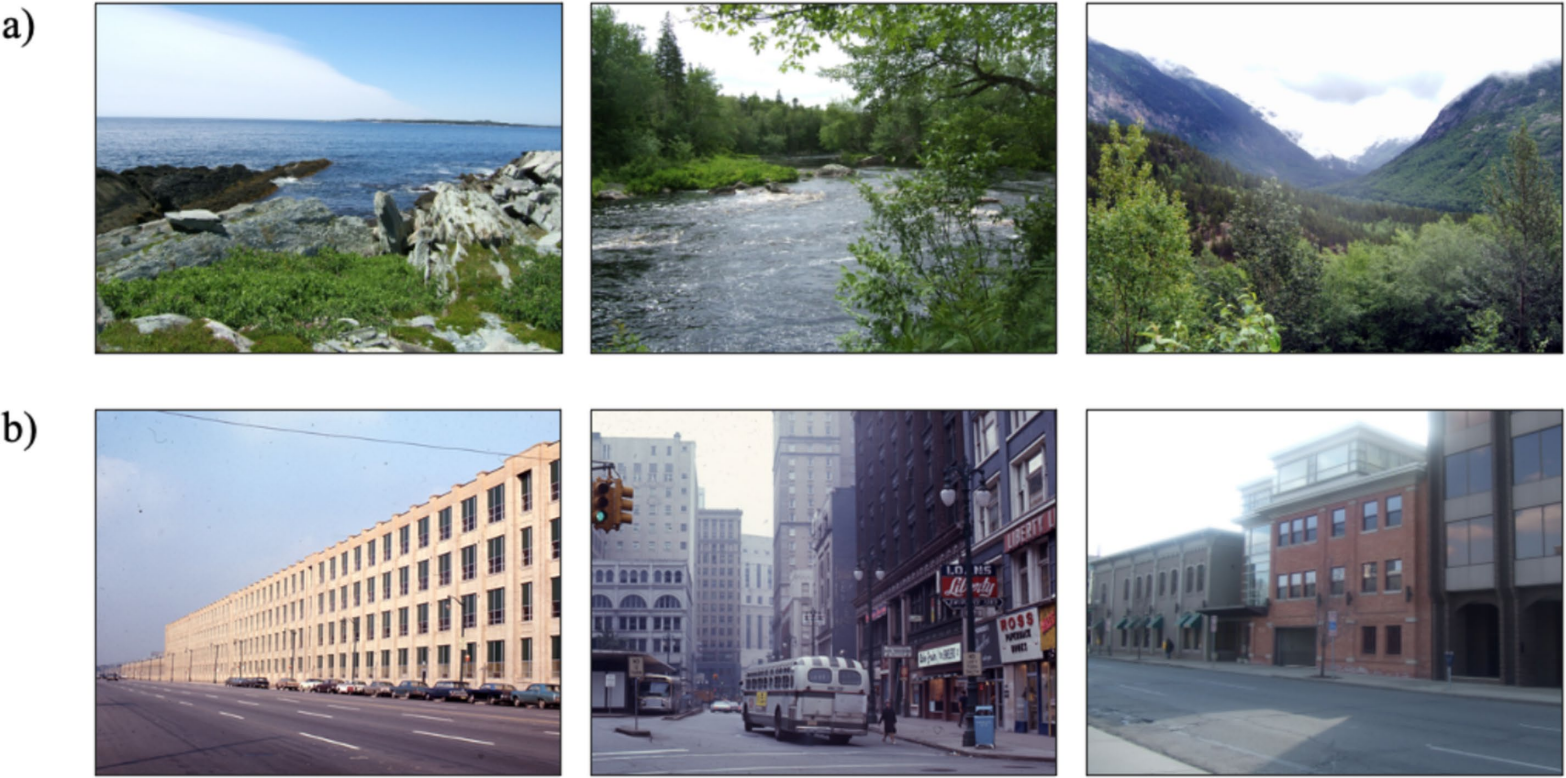
Example stimuli from the nature image set **(a)** and urban image set **(b)**. Images were borrowed from Berman et al. (2008).

Image sets comprised of previously validated nature and urban images utilized extensively in prior literature (Berman et al., 2008; see Figure 1 for example stimuli). The image sets comprised of either 50 nature images or 50 urban images. The nature images consisted of bodies of water and vegetation, dominated by natural elements such as trees, grass, rocks, rivers, and distant mountains and absent of human-made structures. The urban images consisted of buildings and vehicles, dominated by urban elements such as architectural structures, paved roads, and transportation infrastructure, with minimal or no visible natural elements. Replicating previous paradigms using nature and urban images, each image was presented for 7 s in a randomized order and participants viewed the images on a repeating loop for a total of 10 min (Berman et al., 2008; Berto, 2005). Images were displayed in color and full-screen on a 13-inch ACER laptop using Microsoft Office PowerPoint. The laptop sat 24 inches from the participants, and they were instructed to simply sit and passively watch the slideshow of images for the entirety of the 10 min while EEG data were continuously recorded. For access to the complete image sets, see Berman et al. (2008).

### EEG recording and processing

EEG data were collected with BIOPAC’s wireless BioNomadix system (https://www.biopac.com/). Three passive, reusable EEG electrodes (Ag/AgCl) manufactured by Natus (https://natus.com/) were placed along critical midline sites where parietal alpha and frontal theta oscillations are known to be maximally observed: frontal (Fz), central (Cz), and parietal (Pz). A ground electrode was placed in the middle of the forehead and a reference electrode was placed on the right mastoid bone behind the ear. Vertical electrooculographic (VEOG) activity generated from blinks was recorded using two additional passive electrodes placed above and below the right eye in line with the center of the pupil. Electrodes were placed in accordance with the International 10–20 system (Jasper, 1958) and the research team exfoliated the skin with exfoliant gel and Q-tips to clear away dead skin cells at each electrode location. Electrode impedances were kept below 10kΩ to ensure adequate signal to noise ratio. Two wireless EEG transmitters recorded the EEG signal, the BioNomadix Smart Center amplified the signal with a 2 kHz per channel maximum sampling rate, and data were observed online through BIOPAC’s AcqKnowledge software (Version 5.0).

EEG data were processed offline in MATLAB using the EEGLab (Delorme and Makeig, 2004) and ERPLab (Lopez-Calderon and Luck, 2014) toolboxes. Data were downsampled to 250 Hz and band pass filtered from 0.1-30 Hz with a Butterworth filter type and a roll-off of 12 dB/octave. Artifacts created by blinks and eye movements were corrected using Gratton’s eye movement correction procedure (EMCP; Gratton et al., 1983). To account for any artifacts left undetected by EMCP, subsequent artifact rejection was performed using a moving window to reject sections of data containing flatlines or peak to peak activity greater than 200 μV (Lopez-Calderon and Luck, 2014). On average, only 0.73% of data was lost after this artifact correction and subsequent rejection procedure: 0.34% in the nature image condition and 1.10% in the urban image condition.

Artifact-free data were epoched into 1-s intervals using a Hanning window. We used a Fast Fourier Transform to convert the artifact-free data from the time domain to the frequency domain, and the average power at each frequency from 1 to 30 Hz was extracted for each EEG recording. The average power from 8–12 Hz at the parietal electrode (Pz) was used to quantify parietal alpha power and average power from 4 to 8 Hz at the central electrode (Cz) was used to quantify frontal theta power. These electrode locations were selected *a priori* based on prior literature (e.g., McDonnell et al., 2023, 2024) and confirmed *post hoc*, as these are the locations where each oscillation was maximally seen in the current study.

### Perceived restorativeness scale (PRS) short version

At the end of the testing session, participants were instructed to rate how restorative they perceived the images that they viewed to be on a 5-item, shortened version of the PRS, with higher values representing greater perceived restoration (Berto, 2005). The five items on the scale were as follows: (1) “That is a place which is away from everyday demands and where I would be able to relax and think about what interests me,” (2) “That place is fascinating; it is large enough for me to discover and be curious about things,” (3) “That is a place where the activities and the items are ordered and organized,” (4) “That is a place which is very large, with no restrictions to movements; it is a world of its own,” and (5) “In that place, it is easy to orient and move around so that I could do what I like.” Potential scores on each item ranged from 1 (Not at All) - 10 (Very Much) and the scores were averaged to obtain a total score for each participant. This measure served as a manipulation check to ensure the effectiveness of the nature versus urban image intervention.

### Statistical analyses

Statistical analyses were performed using custom Python scripts and established statistical libraries including scipy (Virtanen et al., 2020) and numpy (Harris et al., 2020). All data visualizations were created using the matplotlib toolkit (Hunter, 2007).

For both the EEG and self-report data, independent samples *t*-tests were conducted to compare the means of the two experimental conditions (nature images and urban images). Before performing the *t*-test, assumptions were checked for normality and equal variances. Normality was assessed using the Shapiro–Wilk test (*scipy.stats.shapiro*) for both groups, with *p*-values indicating whether the data deviated significantly from a normal distribution. Additionally, the assumption of equal variances between groups was assessed using Levene’s test (*scipy.stats.levene*). If the assumptions were not met, the raw data were log transformed (*numpy.log*) and then re-assessed for assumptions. Once the assumptions were confirmed, a parametric, independent samples t-test (*scipy.stats.ttest_ind*) was performed to determine whether there was a significant difference between the two groups. Cohen’s *d* effect sizes were calculated for all effects and are interpreted based on Cohen’s (1988) guidelines, where 0.2 represents a small effect, 0.5 represents a medium effect, and 0.8 or greater represents a large effect.

## Results

EEG data from three participants were lost due to electrical interference that rendered the EEG data unusable. This type of electrical interference is due to the wireless recording nature of BIOPAC’s BioNomadix system and suggests that either the participant had a cellphone in their pocket or the stimulus presentation computer was situated between the wireless transmitter and the SmartCenter amplifier, causing the EEG signal to intermittently drop due to signal interference. Therefore, the final EEG analyses were conducted on data from 58 participants: 30 in the nature image condition and 28 in the urban image condition. Furthermore, three different participants did not complete the Perceived Restorativeness Scale. Therefore, the final self-report analyses were conducted on data from 58 participants: 28 in the nature image condition and 30 in the urban image condition. In both cases, the final EEG and self-report datasets (*N* = 58) did not drop below the sample size suggested by the *a priori* power analysis (*N* = 52), therefore this attrition does not substantially impact statistical power or threaten the validity of the results.

### Parietal alpha power

**Figure 2:**
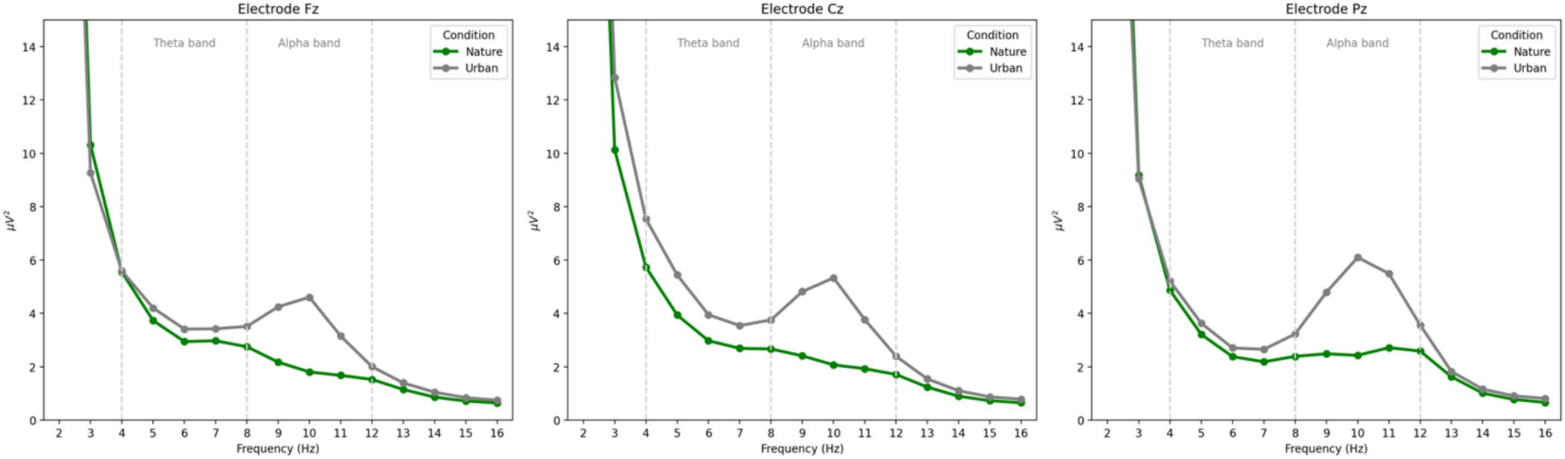
Power spectral density plots of the power at each frequency for each condition at each electrode. Frontal theta power is observed between 4 and 8 Hz and parietal alpha power is observed between 8 and 12 Hz. Consistent with prior literature, frontal theta power was maximal at electrode Cz and parietal alpha power was maximal at electrode Pz.

Power spectral density (PSD) plots depicting the power from 1 to 16 Hz for each experimental condition (nature images and urban images) at each electrode (Fz, Cz, and Pz) are presented in Figure 2.

**Figure 3:**
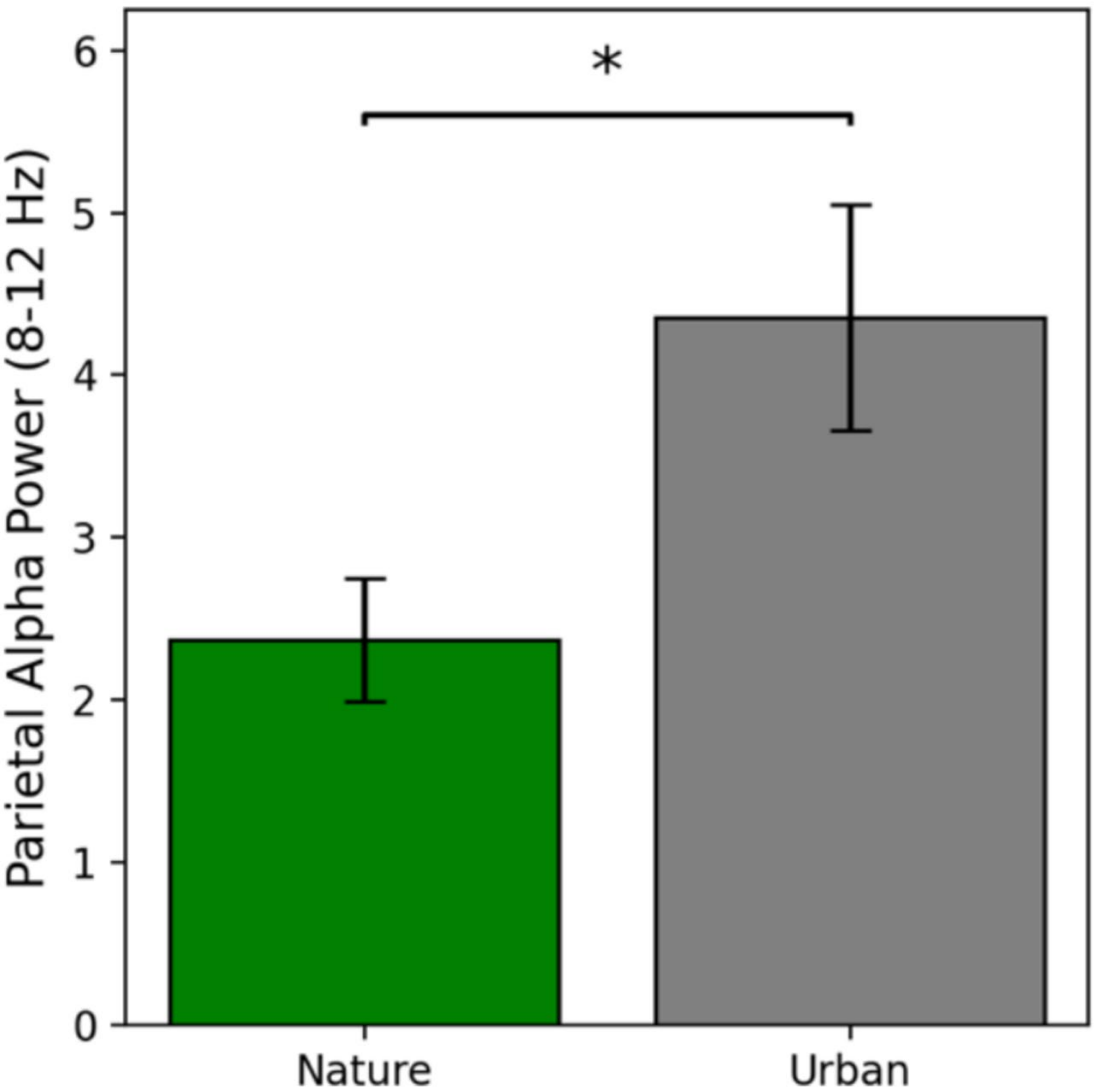
Parietal alpha power as a function of condition at electrode Pz. Error bars represent one standard error of the mean and * indicates a significant effect where *p* < 0.05.

Parietal alpha power is defined as the average power in the alpha band from 8 to 12 Hz at electrode Pz, as this is the electrode where alpha oscillations were maximally seen in this study. Parietal alpha power results are presented in Figure 3.

Parietal alpha power during nature image viewing (*M* = 2.37, *SE* = 0.38) was numerically lower than parietal alpha power during urban image viewing (*M* = 4.36, *SE* = 0.70). Prior to conducting an independent samples *t*-test for significance testing, the assumptions of normality and homogeneity of variances were tested using the Shapiro–Wilk test and Levene’s test, respectively. Both assumptions were violated, so raw data were log transformed and then re-assessed for assumptions, at which point the data met the assumptions for normal distribution and equal variances. Therefore, a parametric *t*-test was run on the log transformed data, revealing a significant difference in parietal alpha power between the nature and urban image viewers [*t*(56) = −2.34, *p* = 0.023, Cohen’s *d* = −0.62] such that parietal alpha power was significantly lower during the nature image viewing compared to the urban image viewing. These results indicate that nature images are more visually engaging than urban images, with a medium to large effect size.

### Frontal theta power

**Figure 4:**
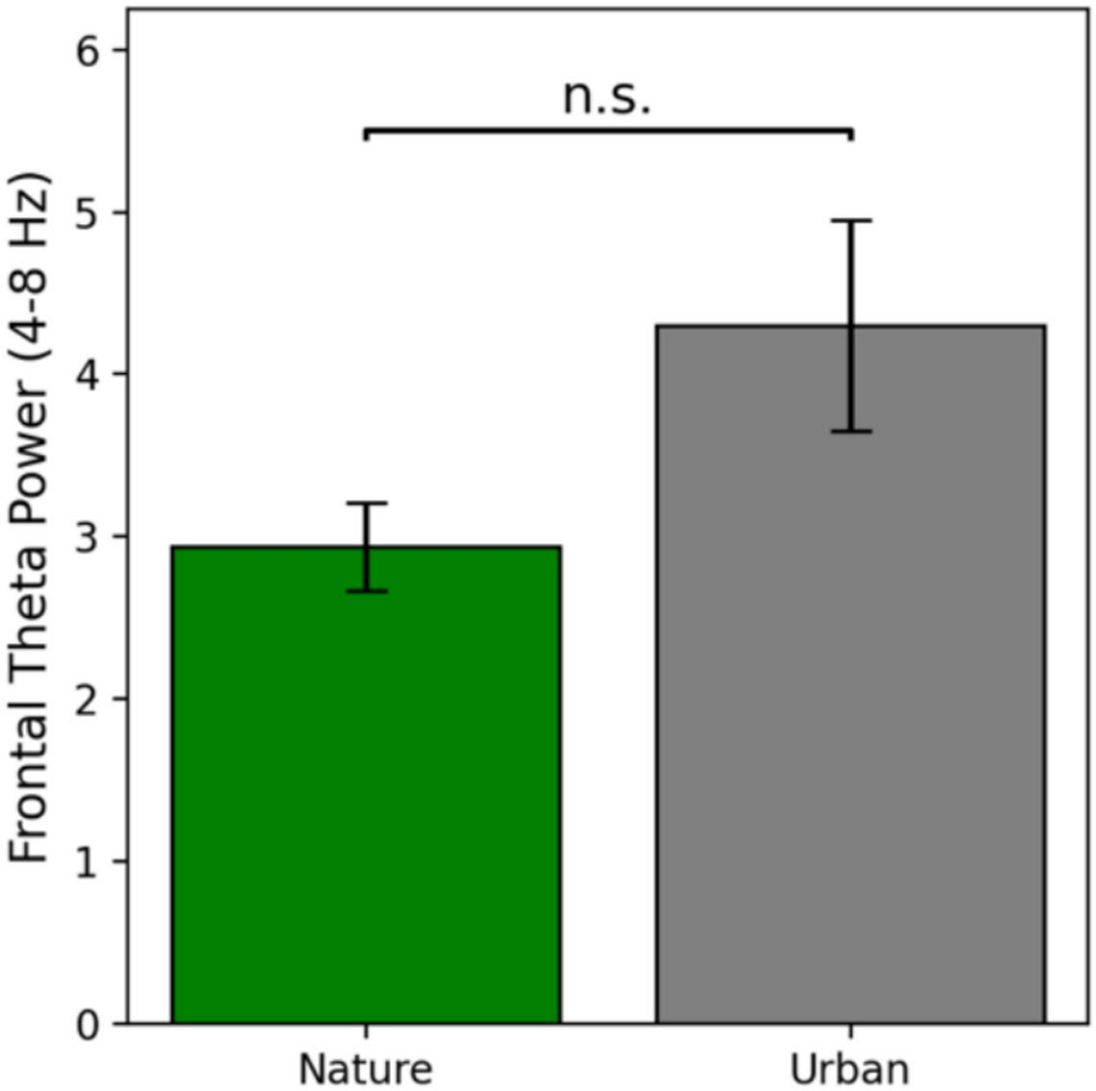
Frontal theta power as a function of condition at electrode Cz. Error bars represent one standard error of the mean.

Frontal theta power is defined as the average power in the theta band from 4 to 8 Hz at electrode Cz, as this is the electrode where theta power was maximally seen in this study. Frontal theta power results are presented in Figure 4.

Frontal theta power during nature image viewing (*M* = 2.94, *SE* = 0.27) was numerically lower than frontal theta power during urban image viewing (*M* = 4.30, *SE* = 0.65). Prior to conducting an independent samples *t*-test for significance testing, the assumptions of normality and homogeneity of variances were tested using the Shapiro–Wilk test and Levene’s test, respectively. Both assumptions were violated, so raw data were log transformed and then re-assessed for assumptions, at which point the data met both assumptions. Therefore, a parametric *t*-test was run on the log transformed data. There was no significant difference in frontal theta power between the nature and urban image viewers [*t*(56) = −1.55, *p* = 0.126, Cohen’s *d* = −0.41]. This suggests that although there was a numeric difference in frontal theta power between viewing nature images and urban images (with a small to medium effect size), there was not a *statistically significant* difference in cognitive effort required to process these different types of images (Figure 4).

### Self-reported perceived restoration

**Figure 5:**
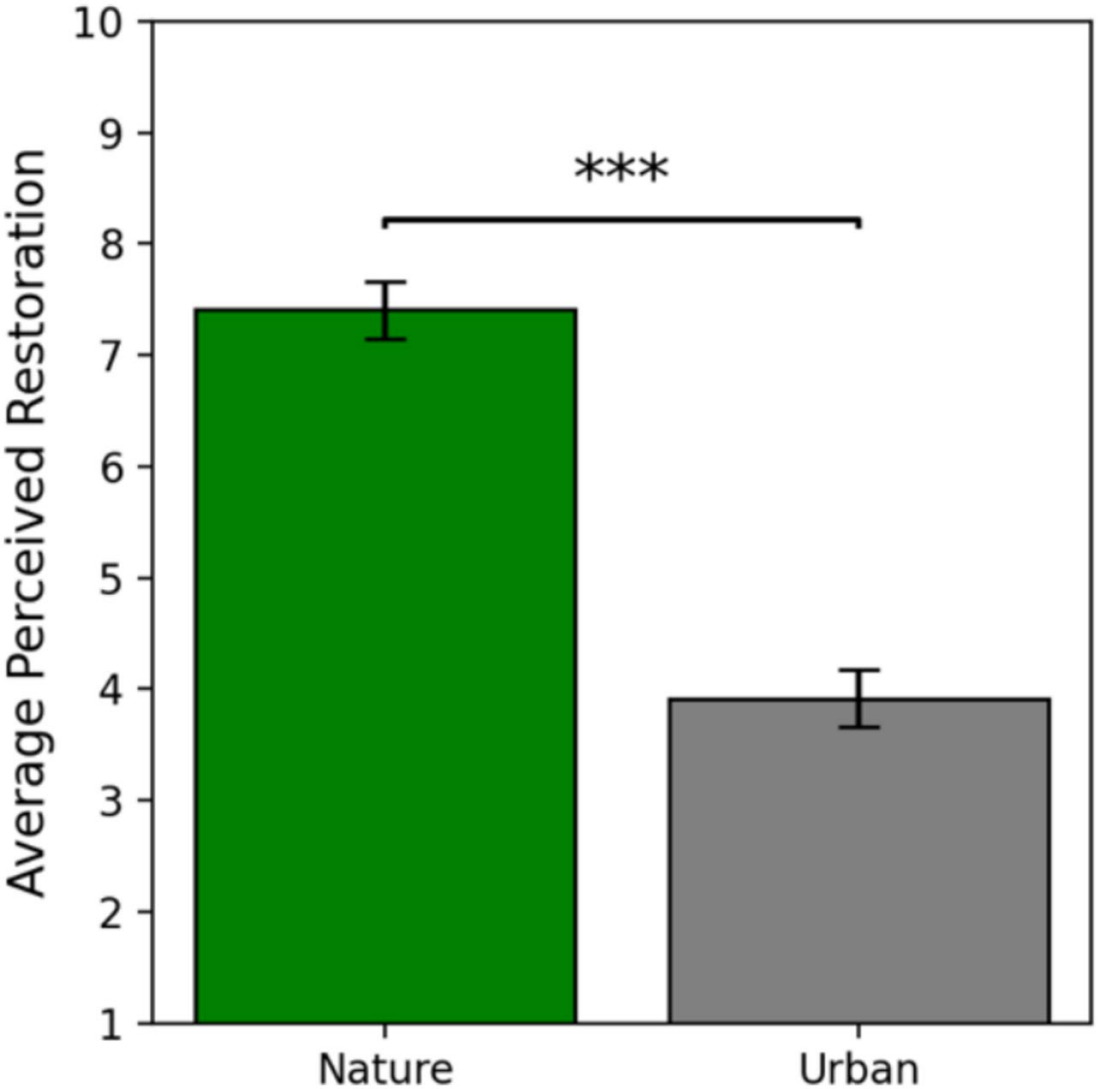
Self-reported perceived restoration as a function of condition. Error bars represent one standard error of the mean and *** indicates a significant effect where *p* < 0.001.

The assumptions of normality and homogeneity of variances of the PRS self-report data were met. Independent samples *t*-tests revealed that participants that viewed the nature images reported their image set to be significantly more restorative (*M* = 7.41, *SE* = 0.26) than the participants that viewed the urban image set [*M* = 3.91, *SE* = 0.26; *t*(56) = 9.58, *p* < 0.001, 95% CI (2.76, 4.23); Cohen’s *d* = 2.51]. These results offer very strong evidence that nature images are perceived to be more restorative than urban images, serving as an effective manipulation check confirming the differential restorative quality of the two image sets (Figure 5).

## Discussion

This study leverages principles of environmental neuroscience to further elucidate the influence of exposure to natural stimuli on brain activity that may underlie and facilitate attention restoration. Specifically, we utilized EEG to explore differences in parietal alpha power and frontal theta power—neural oscillations related to visual engagement and cognitive demand, respectively—between the viewing of nature and urban images. We recorded EEG while participants passively viewed either nature images or urban images for 10 min. Following image viewing, participants self-reported the extent to which they considered their assigned image set to be restorative.

This work is grounded in Attention Restoration Theory (ART; Kaplan, 1995), which posits that natural environments are restorative because they alleviate the attentional demands of modern, urban environments. According to ART, environments must meet four key qualifications to be restorative. First, they must be compatible with an individual’s needs and goals, whether for relaxation, stimulation, or connection. Second, they should provide a sense of being away, allowing mental escape from daily stressors, whether through physical distance or a shift in awareness. Third, a restorative environment must have extent, offering richness and depth that encourage exploration. Finally, it should promote *‘soft fascination’*, where gentle, effortless stimuli—like patterned leaves or rippling water—engage attention without depleting cognitive resources, allowing attentional systems to rest and recover from fatigue. Therefore, according to ART, nature is visually engaging but not in a cognitively demanding fashion.

We hypothesized (*H*_1_) that the nature viewers would display lower parietal alpha power than the urban viewers, suggesting nature images are more visually engaging than urban images. In line with our *a priori* hypothesis, the nature viewers did show significantly lower power in the alpha frequency band distributed over parietal cortices—a reliable marker of visual engagement (Clayton et al., 2018; Peylo et al., 2021; Rana and Vaina, 2014; Romei et al., 2008). These results replicate the findings of Hopman et al. (2020) who found a decrease in parietal alpha power during immersion in nature compared to immersion in an urban environment. It also replicates the Grassini et al. (2019) finding of a decrease in high alpha band activity (11–13 Hz) during the viewing of nature images compared to urban images. Taken together, compounding results across multiple studies offer evidence that nature scenes—whether they are real or images—are more visually engaging than urban scenes.

Alpha oscillations are one of the most explored oscillations in the environmental neuroscience literature, though with mixed results. In a seminal study in the field, Ulrich (1981) measured alpha oscillations during the viewing of nature images, finding increased alpha activity associated with viewing nature images compared to urban images. This pattern has since been replicated by several research teams (Chang et al., 2008; Grassini et al., 2019, 2022). These results are seemingly contradictory to the results of Hopman et al. (2020), who found a *decrease* in alpha power during immersion in nature. It is important to point out two distinctions between these studies that may explain conflicting results. First, Hopman et al. (2020) specifically quantified alpha oscillations over visual cortices (i.e., posterior regions of the scalp), whereas previous studies have quantified alpha over frontal (Chang et al., 2008) and central (Grassini et al., 2019, 2022; Ulrich, 1981) regions. Undoubtedly, alpha oscillations distributed over various cortical regions reflect different cognitive processes, such that parietal alpha has been reliably linked to visual engagement (Clayton et al., 2018; Peylo et al., 2021; Rana and Vaina, 2014; Romei et al., 2008) and frontal/central alpha has been equated to wakeful relaxation in the environmental neuroscience literature. Second, Hopman et al. (2020) quantified alpha power during a multi-day immersion in nature while participants were actively viewing real nature, whereas the other work explores artificial representations of nature such as images or videos.

In the current work, we align with the approach taken by Hopman et al. (2020) and quantify alpha power over parietal cortices as a measure of visual engagement, specifically, as participants view nature and urban images. The inconsistency in results may also be due to differences in low versus high alpha band activity. Grassini et al. (2019) found dissociating effects of nature images on low (8–10 Hz) and high (11 – 13 Hz) alpha such that nature images showed an increase in *low* alpha activity compared to urban images, while nature images showed a decrease in *high* alpha activity compared to urban images. While the spectral curves presented in Figure 2 suggest that both low and high alpha show similar patterns in the current work, these discrepancies in prior work strongly suggest that alpha oscillations are sensitive to electrode location and the specific frequencies an oscillatory band is quantified at; therefore, future research should be particularly sensitive to these methodological considerations.

In terms of frontal theta power, we hypothesized (*H*_2_) that the nature viewers would display lower frontal theta power than the urban viewers, suggesting that visually processing nature images is less cognitively demanding than urban images. While frontal theta power was numerically lower while viewing nature images compared to urban images, there was no statistically significant difference in frontal theta power between the two groups. While not significant, this patten of results replicates the findings of Grassini et al. (2019), who found significantly lower power in the theta band during the viewing of nature images compared to urban images. These results also support the findings of McDonnell and Strayer (2024b) who found significantly lower resting frontal theta power after a nature walk compared to an urban walk. These findings across multiple studies suggest that viewing nature images may be less cognitive demanding than viewing urban images, an idea supported by recent vision science work (Rim et al., 2024). However, due to the lack of a statistically significant result (but rather just a trend in the direction of the hypothesized effect), these findings should be interpreted with caution and replicated in future work.

From a self-report perspective, we hypothesized (*H*_3_) that the nature viewers would rate their image set as more restorative than the urban viewers would. In line with our *a priori* hypothesis, the nature viewers did rate their image set as significantly more restorative on the shortened Perceived Restorativeness Scale (PRS; Berto, 2005) than the urban viewers. This result is consistent with prior research (e.g., McDonnell and Strayer, 2024a) and is one of the most robust findings in the environmental neuroscience literature [for review see Menardo et al. (2021)]. The PRS is a validated scale designed to specifically measure the four qualifications for restoration presented in ART, with questions oriented toward the *compatibility* of the environment, the sense of *being away* offered by the environment, the *extent* of the environment, and the presence of stimuli that engage ‘*soft fascination*’. Thus, these results suggest that to some degree, 2D representations of nature tap into these qualifications enough that participants self-report nature images to be restorative. Therefore, we can conclude that nature images have the necessary components to reduce demands on attention.

Taken together, the results of this study suggest that nature images are perceived as more restorative than urban images. From a neurological standpoint, nature images are perhaps more visually engaging than urban images, but not in a cognitively demanding fashion. These findings align closely with the propositions set forth by ART. Further, these findings support a complementary framework called the Perceptual Fluency Account (Joye and Van den Berg, 2018), which aligns closely with ART in explaining *why* nature images can promote attentional recovery. ART suggests that natural environments help restore attention by providing ‘*soft fascination*’, or gentle, effortless engagement that does not demand cognitive effort. The Perceptual Fluency Account supports this by emphasizing that nature scenes are processed more easily due to their organic patterns, smooth transitions, and fractal structures, which require less cognitive effort compared to the sharp angles and clutter of urban scenes. This ease of processing, or fluency, enhances positive affect and reduces cognitive load, making nature images aesthetically pleasing, visual engaging, and mentally restorative (Joye and Van den Berg, 2018).

### Limitations and future directions

Discussion of the Perceptual Fluency Account brings to the surface important questions about how specific low-level visual features—such as fractal patterns, spatial frequency, hue, and edge density—influence neural processing and subsequent attention restoration. In the current study, these low-level features were not systematically manipulated or isolated. Instead, oscillatory activity was averaged across images with varying visual properties, drawn from pre-existing image sets in the literature (Berman et al., 2008). As a result, the study is limited in its ability to pinpoint which *specific* aspects of nature contribute most to differences in neural oscillations associated with visual engagement and cognitive demand. Future research could systematically compare these features, such as comparing high- and low-fractal images (Joye et al., 2016), varying levels of edge density (Berman et al., 2014), or various spatial frequencies (Thompson et al., 2025; Valtchanov and Ellard, 2015) to determine how each factor uniquely influences perceptual fluency and attention restoration, as proposed by Schertz and Berman (2019). By doing so, we can refine our theoretical frameworks for linking low-level vision, perceptual fluency, attention restoration, and underlying brain activity.

Furthermore, the image sets included in the current study were selected for consistency with prior work, as they have been used for over a decade to study the effects of environmental images on cognitive outcomes (e.g., Charbonneau et al., 2024). However, the images were not normed on properties that may influence visual and cognitive engagement such as arousal and valence (Russell, 1980), mystery (Marois et al., 2021), or preference (Meidenbauer et al., 2020). In recent years, laudable efforts have been made to produce normed image sets on such features (e.g., Meidenbauer et al., 2020; Szolosi et al., 2014). Therefore, future work should replicate the design utilized in the current study while using image sets that are normed for asking questions related to arousal, engagement, and cognitive demand.

This study was theoretically grounded in ART, therefore the selection of neural oscillations measured, and the interpretation of results, lean heavily upon theories of attention. However, it is possible that there are other physiological mechanisms at play that may influence the results and interpretation of this work. For example, it is possible that there are differences in stress associated with viewing nature and urban images. This proposition is supported by another seminal theory in the environmental neuroscience literature—Stress Recovery Theory (SRT; Ulrich et al., 1991)—which suggests that nature promotes recovery from stress. Although we did not measure or manipulate stress directly in this study, it is possible that stress recovery is co-occurring alongside attention restoration, a concept supported by a unified framework presented by Scott et al. (2021). In other words, there may be some degree of changes in stress that could influence the mechanisms that underlie restoration in nature. It is also important to acknowledge the limitations of reverse inference, which are inherent in all psychophysiology studies (e.g., Nathan and Del Pinal, 2017; Poldrack, 2008). While previous research has linked the neural oscillations examined in this study to visual engagement and cognitive demand, we cannot rule out the possibility that there are other psychological processes at play that are contributing to the observed changes. Future studies employing converging methods may be needed to further validate these claims.

Another exciting avenue for future environmental neuroscience research would be to systematically vary both the type and duration of natural stimuli to assess how different aspects of exposure influence neural oscillations related to attention. Little is known about the optimal characteristics and timeframes needed to drive neural changes. For example, researchers could directly compare static images, dynamic videos, and immersive virtual reality representations of the same natural environment to manipulate the extent, or immersiveness, of an environment while holding constant the low-level visual features. Additionally, examining short versus prolonged exposure could provide insight into whether brief encounters with nature—such as viewing a nature image for just a few seconds—can induce measurable changes in neural activity or if longer exposure—such as the 10 min utilized in this study—is required for significant changes in the brain. Investigating the interplay between image complexity and duration could refine our understanding of how the brain processes natural environments and help design more effective interventions for enhancing attention in urbanized settings.

Furthermore, the current study focuses solely on the visual features of nature, limiting our ability to understand how other sensory modalities—such as sound, smell, or touch—may interact with vision to drive differences in neural oscillations related to attention. Future research should explore the multisensory dimensions of ART, investigating whether combined sensory exposure (e.g., viewing nature while listening to natural sounds) leads to greater cognitive benefits compared to visual stimuli alone (Bratman et al., 2024). This could have significant implications for designing restorative environments and alternative interventions such as virtual reality that may serve as effective substitutes. Greater emphasis should also be placed on real-world environmental neuroscience, which can leverage mobile EEG or fNIRS to measure brain activity during multisensory immersion in natural settings (e.g., Hopman et al., 2020; LoTemplio et al., 2020; McDonnell et al., 2025) rather than relying solely on laboratory-based interactions with stimuli that typically engage only one or two senses at a time.

Finally, the current study lacks sufficient statistical power to examine individual differences in response to environmental images. It is possible that individuals with greater experience in natural environments exhibit more pronounced restorative benefits when viewing nature scenes, whereas those who are more accustomed to urban environments might find urban scenes more restorative due to familiarity or learned associations. Harrison (2023) demonstrated that one’s reported connectedness to nature significantly influences the amount of attention an individual allocates to nature scenes as well as their affective response to those scenes. Therefore, it is possible that individual differences in connectedness to nature may differentially influence participants’ neural response to different image sets. Future studies with larger and more diverse samples are needed to investigate how personal background and environmental exposure history may moderate the restorative impact of different environments.

## Conclusion

This study took a hypothesis-driven approach to quantifying neural oscillations related to visual engagement (parietal alpha power) and cognitive demand (frontal theta power) during the viewing of nature and urban images. Our findings provide evidence that nature images promote visual engagement without imposing excessive cognitive load, aligning closely with the theoretical propositions of Attention Restoration Theory and the Perceptual Fluency Account. Moreover, given that self-reported perceived restoration was significantly higher for nature images, our results suggest that even 2D representations of nature can evoke a sense of restoration. These results contribute to the growing body of environmental neuroscience research, supporting the idea that exposure to nature images differentially influences brain activity and feelings of restoration when compared to urban images. While this study provides valuable insights into the neural mechanisms underlying nature’s restorative benefits, it raises important questions regarding the role of other sensory modalities, the optimal duration of exposure, the role of individual differences, and potential interactions with stress recovery processes. By integrating neurological, psychological, and environmental perspectives, future research can deepen our understanding of how natural environments contribute to cognitive well-being and inform interventions designed to counteract the demands of modern urban life.

## Data Availability

The raw data supporting the conclusions of this article will be made available by the authors, without undue reservation.
